# Dolphins simplify their vocal calls in response to increased ambient noise

**DOI:** 10.1098/rsbl.2018.0484

**Published:** 2018-10-24

**Authors:** Leila Fouda, Jessica E. Wingfield, Amber D. Fandel, Aran Garrod, Kristin B. Hodge, Aaron N. Rice, Helen Bailey

**Affiliations:** 1 Chesapeake Biological Laboratory, University of Maryland Center for Environmental Science, Solomons, MD, USA; 2 Bioacoustics Research Program, Cornell Laboratory of Ornithology, Cornell University, Ithaca, NY, USA

**Keywords:** acoustic communication, anthropogenic noise, bottlenose dolphin, vocal modification

## Abstract

Ocean noise varies spatially and temporally and is driven by natural and anthropogenic processes. Increased ambient noise levels can cause signal masking and communication impairment, affecting fitness and recruitment success. However, the effects of increasing ambient noise levels on marine species, such as marine mammals that primarily rely on sound for communication, are not well understood. We investigated the effects of concurrent ambient noise levels on social whistle calls produced by bottlenose dolphins (*Tursiops truncatus*) in the western North Atlantic. Elevated ambient noise levels were mainly caused by ship noise. Increases in ship noise, both within and below the dolphins' call bandwidth, resulted in higher dolphin whistle frequencies and a reduction in whistle contour complexity, an acoustic feature associated with individual identification. Consequently, the noise-induced simplification of dolphin whistles may reduce the information content in these acoustic signals and decrease effective communication, parent–offspring proximity or group cohesion.

## 1. Introduction

Ambient noise levels vary spatially and temporally and are affected by numerous activities and processes, both natural and anthropogenic [1]. Increased ambient noise levels can reduce the ability of animals to perceive acoustic signals (masking) and have been associated with alterations in animal vocalizations (e.g. [2]) as well as negative impacts on health and reproduction [3,4]. Vocal communication plays a critical role in many species, such as in parent–offspring interactions, warning calls, mating signals and territorial defence. Vocal adjustments may compensate for increased ambient noise, but there may be constraints that limit this ability [5] or ecological consequences to modified signals. If communication is impaired, this may lead to behavioural changes, which can affect fitness and recruitment [6].

Odontocetes have complex social structures that are probably maintained through their diverse and individually specific vocalizations [7]. One of the best-studied odontocete species, the bottlenose dolphin (*Tursiops truncatus*), produces whistles that serve a critical role in social communication, conveying individual identity and other information through contour shape [8]. Vessel traffic and noise have been found to affect marine mammal foraging behaviour [9–11] and the sound frequency of their calls [12,13]. However, little is known about how the complexity of their calls changes in response to real-time ambient noise levels experienced by the animals. We addressed this by investigating whether the acoustic characteristics of bottlenose dolphin whistles changed in response to concurrent ambient noise levels (both from natural and anthropogenic sources). Our study area in the northwest Atlantic Ocean experiences relatively high levels of vessel traffic that we hypothesized would result in regularly elevated noise conditions and could consequently impact dolphin call patterns.

## 2. Methods

### (a) Data collection and analysis

Acoustic recordings were collected using a bottom-mounted SM3M recorder (Wildlife Acoustics) sampling at 48 kHz during July–September 2016, located approximately 30 km offshore of Maryland, USA, in the western North Atlantic Ocean (electronic supplementary material, figure S1). Spectrograms were visually inspected for bottlenose dolphin whistles with high signal-to-noise ratios [12] in Raven Pro (v. 1.5). For each whistle selected, 11 characteristics were measured: duration; start and end frequencies; minimum, maximum and delta frequency (maximum–minimum frequency); the presence of harmonics, and number of extrema, inflection points, saddles and steps (electronic supplementary material, figures S2 and S3).

Ambient noise levels were calculated for the 2 s period prior to selected whistles [14]. PAMGUARD‘s Noise Monitor Module was used to measure root-mean-square (RMS) sound pressure levels in both the broadband signal (2 Hz–24 kHz) and one-third octave band levels (TOLs) centred on frequencies from 12.5 Hz to 20 kHz. Ambient noise levels for each 2 min recording across the entire deployment period were also calculated to determine how frequently relatively high noise levels (greater than 120 dB re 1 µPa RMS, the USA marine mammal regulatory threshold for behavioural disruption from continuous noise) occurred.

### (b) Statistics

The effect of ambient noise levels at each frequency band was tested on the suite of whistle characteristics using a multivariate analysis of variance (MANOVA). Generalized estimating equations (GEEs) were then fitted with each whistle characteristic as the response variable and the suite of ambient noise levels that were statistically significant in the MANOVAs as the explanatory variables. The encounter identification number (where an encounter consisted of continuous detections) was treated as the cluster grouping with an exchangeable working correlation structure. A Holm–Bonferroni sequential correction for multiple tests was applied [15].

## 3. Results

**Figure 1.**
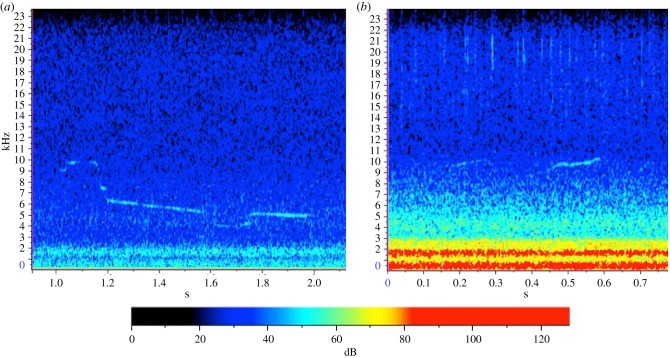
Spectrograms of example whistle during (*a*) relatively low ambient noise (108.2 dB re 1 µPa) on 14 September 2016, and (*b*) relatively high ambient noise (133.6 dB re 1 µPa) on 7 September 2016.

**Figure 2.**
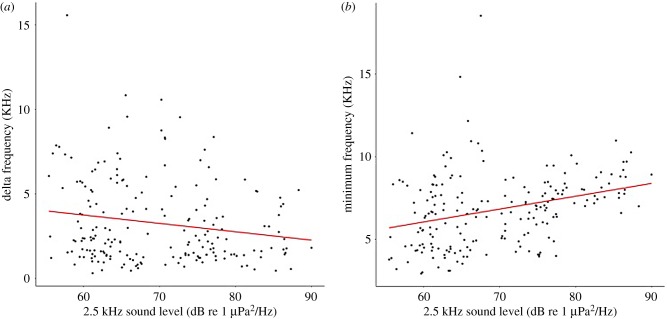
Effect of 2.5 kHz TOL on (*a*) delta frequency and (*b*) minimum frequency of dolphin whistles with linear regression lines. (Online version in colour.)

In total, 200 high-quality whistles from 16 encounters were used in the analysis (figure 1, [16]). Whistles occurred in the frequency range 2.93–23.83 kHz (mean 6.79–10.08 kHz) with durations of 0.07–1.17 s (electronic supplementary material, table S1). Ambient broadband noise associated with these whistles was 108.1–134.2 dB re 1 µPa (64.4–90.3 dB re 1 µPa^2^ Hz^−1^) and had a significant effect on whistle characteristics (MANOVA: *F*_12_ = 2.7, *p* < 0.01). Increased noise in the 2.5 kHz TOL significantly affected the greatest number of characteristics, including reducing whistle length, delta frequency and the number of steps while increasing the start and minimum frequency (table 1 and figure 2). A significant reduction in the number of inflections and saddles occurred during increased ambient noise in the 20 kHz TOL. Increased noise in the 40 Hz, 400 Hz and 10 kHz TOLs also had significant effects on dolphin whistle characteristics (table 1). Over the entire deployment period, relatively high ambient noise levels were mainly caused by vessel noise and were above 120 dB re 1 µPa 11% of the time (electronic supplementary material, figures S4 and S5).

**Table 1.** Statistically significant results from the GEE models. (TOL refers to third octave band levels.)

frequency band	response variable	estimate	s.e.	Wald	Pr(>|W|)
broadband	minimum frequency	94.80	20.10	22.30	<0.01
	maximum frequency	56.40	24.50	5.28	0.02
	start frequency	91.50	24.00	14.54	<0.01
40 Hz TOL	extrema	−0.08	0.020	15.20	<0.01
400 Hz TOL	delta frequency	43.00	14.10	9.37	<0.01
	steps	0.07	0.02	8.14	<0.01
	saddles	−0.02	0.01	12.11	<0.01
2.5 kHz TOL	length	−0.005	0.001	14.14	<0.01
	minimum frequency	86.52	22.16	15.25	<0.01
	delta frequency	−67.20	24.10	7.75	<0.01
	start frequency	61.60	22.80	7.31	<0.01
	harmonics	−0.007	0.003	5.35	0.02
	steps	−0.04	0.02	6.69	0.01
	saddles	0.03	0.01	9.29	<0.01
10 kHz TOL	saddles	0.09	0.02	20.97	<0.01
20 kHz TOL	inflections	−0.10	0.05	4.32	0.04
	saddles	−0.05	0.02	8.27	<0.01

## 4. Discussion

Bottlenose dolphins change their vocalization characteristics during increased ambient noise. Such changes have also been observed in primates, birds, bats and other species to counteract masking effects [3] and this is emerging as a widespread response to elevated ambient noise. In our study, we specifically examined the ambient noise level immediately prior to the call and examined contour shape characteristics as well as frequency parameters of the dolphin whistles. The dolphin whistles had a less complex contour shape during increased ambient noise in the 2.5 and 20 kHz TOLs. Since these frequencies are outside the mean range of the whistles, this suggests that the signaller responded by modifying the call as opposed to the received call losing components through masking. These modifications may serve to simplify the call, thereby reducing the potential loss of information owing to masking by ambient noise. Beluga whales (*Delphinapterus leucas*) in the St Lawrence River similarly produced less frequency modulated calls when background noise became louder owing to vessel noise [17]. Call duration compression may serve to fit calls into quieter intervals [18]. It is unknown what impact this shortening and simplification of calls may have on the information communicated. There are, to our knowledge, currently no studies that have addressed the call receivers to determine if and how call simplification may affect dolphin fitness. Vocal communication is important in dolphin mother–offspring interactions and social bonding [7]. The frequency modulation pattern of calls carries identity [8] and other information, and consequently there could be changes to the level of information communicated if individuals respond to increased ambient noise by simplifying the features of their whistles. The ambient noise environment could also affect vocal learning, as young animals exposed to elevated noise may hear adjusted calls from conspecifics [19].

In addition to modifying the shape, dolphin whistles were also higher frequency during increased broadband and 2.5 kHz TOL ambient noise immediately prior to the call. Marine mammals have been recorded increasing the amplitude [20,21], altering the frequency parameters [12,14] or call rate [22] of their calls in response to ambient noise. Masking occurs when ambient noise overlaps with the frequency band of the calls (energetic masking), but can also occur when signals cannot be perceptually distinguished from other noise (informational masking) [23]. Dolphins adjusted their calls when noise levels were elevated at a range of frequencies, including below the frequencies of their whistle calls. Increased low-frequency ambient noise may be causing dolphins to change their vocalization behaviour to avoid or compensate for masking. These changes could be detrimental to conspecific communication and potentially reduce group cohesion as has been found in terrestrial species [3,6].

Although ambient noise may increase as a result of natural processes, elevated noise conditions in our study were primarily attributed to vessel noise. The study area is adjacent to the shipping lanes into Delaware Bay (electronic supplementary material, figure S1) and had ambient noise levels comparable to other coastal areas with frequent vessel traffic [24]. If a vessel is located in a different direction from conspecifics, there may be a decrease in masking (spatial release from masking) [23], but the observed changes in the whistle signals indicate that the dolphins simplified their calls to counter the masking effects of vessel noise. Although marine mammals demonstrate vocal plasticity, there may be constraints to their vocal compensatory capabilities and its sustainability over time. Ambient noise levels are likely to rise in the future as vessel traffic increases and an offshore wind energy facility is proposed. Regulations and voluntary incentives to reduce the sound production of vessels, for example with speed limits or quieter engines, could help to decrease the effects on dolphins and other species sensitive to sound.

## Supplementary Material

Supplemental Methods and Figures

## Data accessibility

The datasets are available in the Dryad Digital Repository: http://dx.doi.org/10.5061/dryad.t530ps6 [16].

## References

[1] Hildebrand JA 2009 Anthropogenic and natural sources of ambient noise in the ocean. Mar. Ecol. Progress Series 395, 5–20. (10.3354/meps08353)

[2] Slabbekoorn H, Peet M 2003 Birds sing at a higher pitch in urban noise. Nature 424, 267 (10.1038/424267a) 12867967

[3] Barber JR, Crooks KR, Fristrup KM 2010 The costs of chronic noise exposure for terrestrial organisms. Trends Ecol. Evol. 25, 180–189. (10.1016/j.tree.2009.08.002) 19762112

[4] Kight CR, Swaddle P 2011 How and why environmental noise impacts animals: an integrative, mechanistic review. Ecol. Lett. 14, 1052–1061. (10.1111/j.1461-0248.2011.01664.x) 21806743

[5] Nemeth E, Pieretti N, Zollinger SA, Geberzahn N, Partecke J, Miranda AC, Brumm H 2013 Bird song and anthropogenic noise: vocal constraints may explain why birds sing higher-frequency songs in cities. Proc. R. Soc. B 280, 20122798 (10.1098/rspb.2012.2798) PMC3574330 23303546

[6] Schroeder J, Nakagawa S, Cleasby IR, Burke T 2012 Passerine birds breeding under chronic noise experience reduced fitness. PLoS ONE 7, e39200 (10.1371/journal.pone.0039200) 22808028 PMC3394753

[7] Connor RC, Mann J, Tyack PL, Whitehead H 1998 Social evolution in toothed whales. Trends Ecol. Evol. 13, 228–232. (10.1016/S0169-5347(98)01326-3) 21238276

[8] Janik VM, Sayigh LS, Wells RS 2006 Signature whistle shape conveys identity information to bottlenose dolphins. Proc. Natl Acad. Sci. USA 103, 8293–8297. (10.1073/pnas.0509918103) 16698937 PMC1472465

[9] Blair HB, Merchant ND, Friedlaender AS, Wiley DN, Parks SE 2016 Evidence for ship noise impacts on humpback whale foraging behaviour. Biol. Lett. 12, 20160005 (10.1098/rsbl.2016.0005) 27512131 PMC5014013

[10] Wisniewska DM, Johnson M, Teilmann J, Siebert U, Galatius A, Dietz R, Madsen PT 2018 High rates of vessel noise disrupt foraging in wild harbour porpoises (*Phocoena phocoena*). Proc. R. Soc. B 285, 20172314 (10.1098/rspb.2017.2314) PMC5829196 29445018

[11] Pirotta E, Merchant ND, Thompson PM, Barton TR, Lusseau D 2015 Quantifying the effect of boat disturbance on bottlenose dolphin foraging activity. Biol. Conserv. 181, 82–89. (10.1016/j.biocon.2014.11.003)

[12] Heiler J, Elwen SH, Kriesell HJ, Gridley T 2016 Changes in bottlenose dolphin whistle parameters related to vessel presence, surface behaviour and group composition. Anim. Behav. 117, 167–177. (10.1016/j.anbehav.2016.04.014)

[13] van Ginkel C, Becker DM, Gowans S, Simard P 2017 Whistling in a noisy ocean: bottlenose dolphins adjust whistle frequencies in response to real-time ambient noise levels. Bioacoustics 27, 391–405. (10.1080/09524622.2017.1359670)

[14] Marley SA, Salgado Kent CP, Erbe C, Parnum IM 2017 Effects of vessel traffic and underwater noise on the movement, behaviour and vocalisations of bottlenose dolphins in an urbanised estuary. Sci. Rep. 7, 13437 (10.1038/s41598-017-13252-z) 29044128 PMC5647363

[15] Holm S 1979 A simple sequentially rejective multiple test procedure. Scand. J. Stat. 6, 65–70.

[16] Fouda L, Wingfield JE, Fandel AD, Garrod A, Hodge KB, Rice AN, Bailey H 2018 Data from: Dolphins simplify their vocal calls in response to increased ambient noise *Dryad Digital Repository*. (10.5061/dryad.t530ps6) PMC6227850 30355679

[17] Lesage V, Barrette C, Kingsley MCS, Sjare B 1999 The effect of vessel noise on the vocal behavior of belugas in the St. Lawrence River Estuary, Canada. Mar. Mamm. Sci. 15, 65–84. (10.1111/j.1748-7692.1999.tb00782.x)

[18] Egnor SER, Wickelgren JG, Hauser MD 2007 Tracking silence: adjusting vocal production to avoid acoustic interference. J. Comp. Physiol. A 193, 477–483. (10.1007/s00359-006-0205-7) 17242881

[19] Janik VM, Slater PJB 2000 The different roles of social learning in vocal communication. Anim. Behav. 60, 1–11. (10.1006/anbe.2000.1410) 10924198

[20] Holt MM, Noren DP, Veirs V, Emmons CK, Veirs S 2009 Speaking up: killer whales (*Orcinus orca*) increase their call amplitude in response to vessel noise. J. Acoust. Soc. Am. 125, EL27 (10.1121/1.3040028) 19173379

[21] Parks SE, Johnson M, Nowacek D, Tyack PL 2011 Individual right whales call louder in increased environmental noise. Biol. Lett. 7, 33–35. (10.1098/rsbl.2010.0451) 20610418 PMC3030867

[22] Di Iorio L, Clark CW 2010 Exposure to seismic survey alters blue whale acoustic communication. Biol. Lett. 6, 51–54. (10.1098/rsbl.2009.0651) 19776059 PMC2817268

[23] Clark CW, Ellison WT, Southall BL, Hatch L, Van Parijs SM, Frankel A, Ponirakis D 2009 Acoustic masking in marine ecosystems: intuitions, analysis, and implication. Mar. Ecol. Progress Series 395, 201–222. (10.3354/meps08402)

[24] Merchant ND, Pirotta E, Barton TR, Thompson PM 2014 Monitoring ship noise to assess the impact of coastal developments on marine mammals. Mar. Pollut. Bull. 78, 85–95. (10.1016/j.marpolbul.2013.10.058) 24279956

