# Supplementary material for: Dolphins simplify their vocal calls in response to increased ambient noise
Source: Biol Lett. 2018 Oct 24;14(10):20180484. doi: 10.1098/rsbl.2018.0484 (PMC6227850; doi:10.1098/rsbl.2018.0484)
Supplement: Supplemental Methods and Figures [file rsbl20180484supp1.docx]

**Dolphins simplify their vocal calls in response to increased ambient noise**

Leila Fouda, Jessica E. Wingfield, Amber D. Fandel, Aran Garrod, Kristin B. Hodge, Aaron N. Rice, and Helen Bailey

# **Supplementary Material**

Acoustic recordings were obtained from an SM3M acoustic recorder approximately 30 km offshore of Maryland, U.S.A., within the proposed Maryland Wind Energy Area in the Western North Atlantic Ocean (Fig. S1). The acoustic recorder was duty-cycled at 2 minutes on/4 minutes off to increase duration of the recording time given the battery limitations.

The quality of identified whistles were ranked to determine which were considered “high quality”. It was necessary that the whistles were “high quality” and distinct enough from the background noise that their characteristics could be accurately measured. This required an entire whistle being clear on a spectrogram and having a good signal to noise ratio (SNR). We utilized the three-grade system developed by Heiler et al. [1], where levels 2 and 3 were considered “high quality” and included in further analysis:

1. Signal is faint but visible on the spectrogram;
2. Signal is clear and unambiguous;
3. Signal is prominent and dominates.

Additionally, in order to accurately measure the background noise there needed to be a section of the recording immediately prior to a whistle. The PAMGUARD software was calibrated with the settings used on the SM3M hydrophone during recording (hydrophone sensitivity= -165 dB re 1V/µPa, preamplifier gain= 12dB, and peak-peak voltage range = 1.9997 V). Background noise measurements were made every second using PAMGUARD and then averaged across the two-second period. The following statistics were reported: mean, median, min, max, lower 95% level, and upper 95% level. These measurements were taken across the broadband scale (2 Hz - 24 kHz), and also in TOLs centred from 12.5 Hz to 20 kHz to better identify the frequency bands that most overlap, and are most likely to impact dolphin whistles. Eleven whistle characteristics were measured: whistle length, start and end frequency, minimum, maximum, and delta frequency (maximum – minimum frequency), number of extrema (first derivative of the whistle = 0), number of inflection points (second derivative of the whistle = 0), number of saddles (multiple derivatives of the whistle = 0), number of steps, and presence of harmonics (Table S1 and Fig. S2 and S3).

Spectrum density levels (SDL) were calculated to compare between different TOLs and broadband noise using the formula below, in which the received sound level is in dB, ∆f is the difference between the bandwidths’ upper and lower limits, and the SDL units are dB re 1µPa^2^/Hz.

$$SDL=ReceivedSoundLevel-10L{og}_{10}\Delta f$$

To evaluate whistle characteristics from as many dolphins as possible within the population, whistles were selected from multiple days and encounters, where a new encounter occurred when there was >37 minutes between detections (based on the distribution of detections across the deployment period). Whistle characteristics were measured and recorded from spectrogram inspection using Raven Pro 1.5 (Cornell Laboratory of Ornithology, NY). The selected high quality whistles spanned the range of ambient noise levels recorded (Fig. S4 and S5).

High levels of collinearity between TOLs were found when assessed using variance inflation factors (VIFs). Therefore, all TOLs with VIFs > 3 were removed. The bands centered on 40 Hz, 500 Hz, 2500 Hz, 10000 Hz, and 20000 Hz remained, and GEEs were fit incorporating these TOLs as explanatory variables. The error distribution for all of the GEE models was Gaussian with an identity link function. Model fit was assessed by examining plots of fitted values and residuals [2]. All statistical analyses were conducted in the software R [3] and the Generalized Estimating Equations (GEEs) were fit using the geepack package in R [4].

References

[1] Heiler, J., Elwen, S.H., Kriesell, H.J. & Gridley, T. 2016 Changes in bottlenose dolphin whistle parameters related to vessel presence, surface behaviour and group composition. Animal Behaviour 117, 167-177.

[2] Hardin, J.W. & Hilbe, J.M. 2003 Generalized estimating equations. Boca Raton, FL, Chapman and Hall; 222 p.

[3] RStudio Team (2015) RStudio: Integrated Development for R. RStudio, Inc., Boston, MA, http://www.rstudio.com.

[4] Halekoh, U., Højsgaard, S., & Yan, J. (2006) The R Package geepack for Generalized Estimating Equations. Journal of Statistical Software, 15: 1—11.

Table S1: A summary of the mean, minimum, and maximum values for whistle characteristics

| **Whistle Characteristic** | **Mean** | **Minimum** | **Maximum** |
| --- | --- | --- | --- |
| **Length** | 0.4047 s | 0.0670 s | 1.170 s |
| **Minimum frequency** | 6792 Hz | 2929 Hz | 18531 Hz |
| **Maximum frequency** | 10075 Hz | 5081 Hz | 23826 Hz |
| **Delta frequency** | 3282.3 Hz | 310.5 Hz | 15573.8 Hz |
| **Start frequency** | 7808 Hz | 3003 Hz | 19478 Hz |
| **End frequency** | 8958 Hz | 2929 Hz | 19074 Hz |


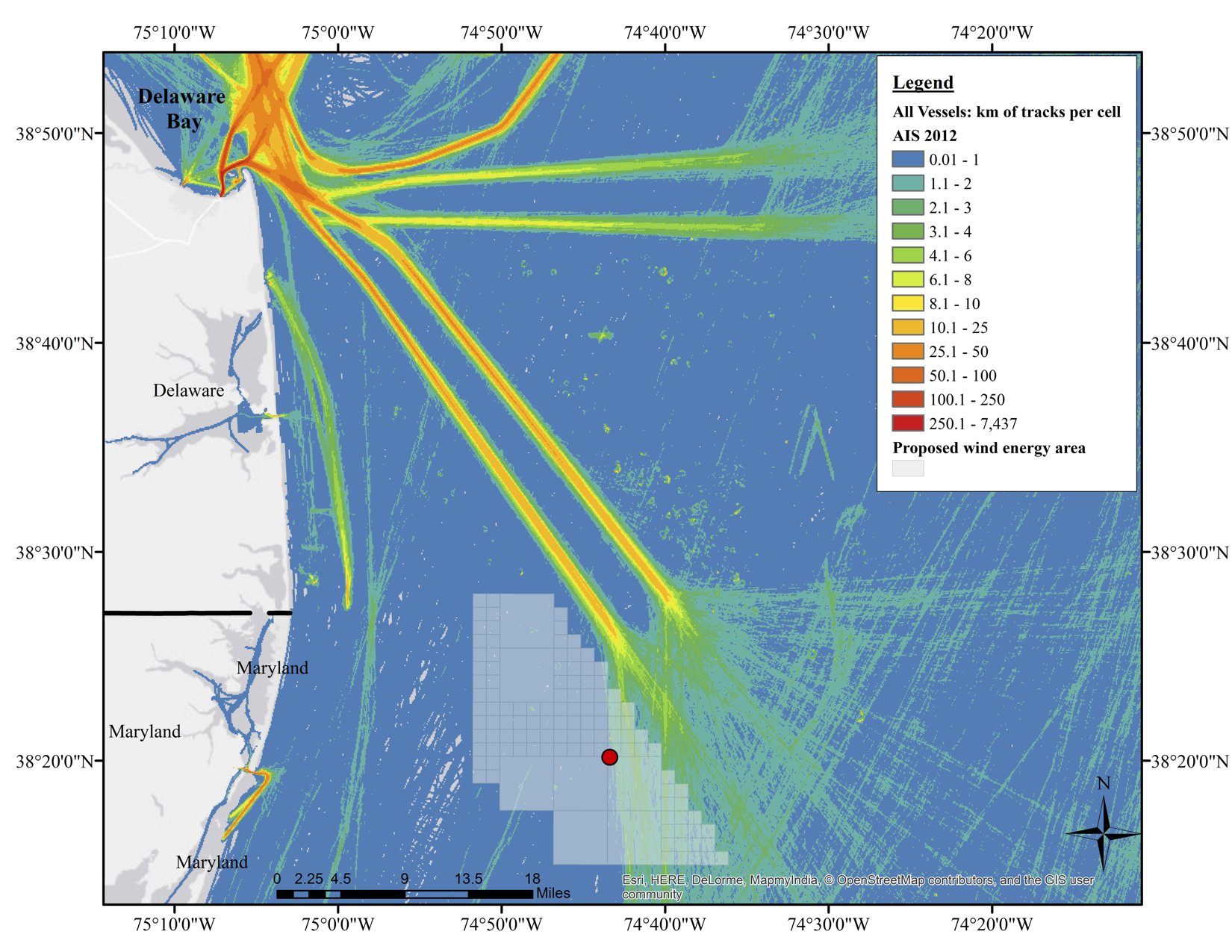

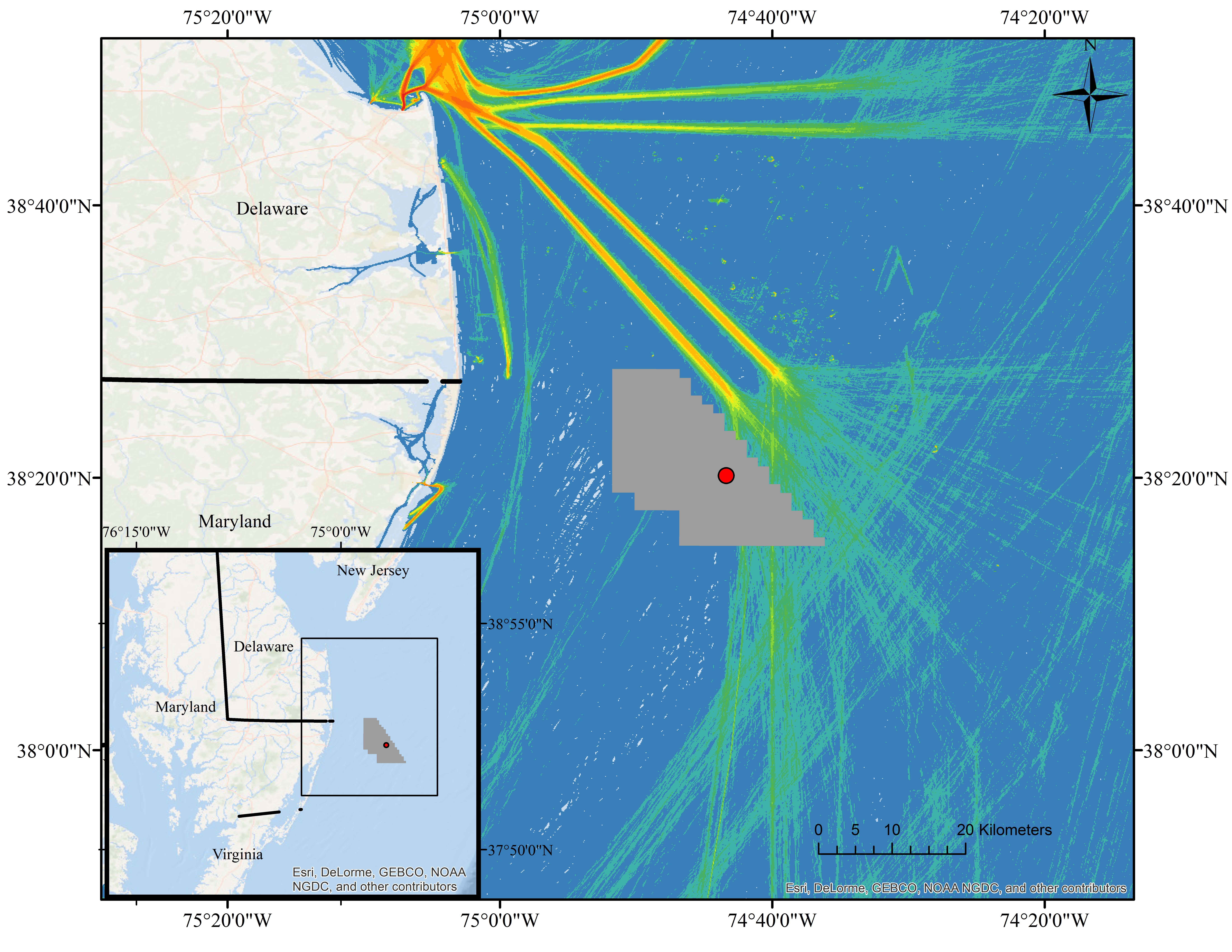


Figure S1: Map of proposed wind energy area (shaded grey blocks), density of commercial vessel traffic (from automatic identification system (AIS) data for 2012 from marinecadastre.gov), and our hydrophone (red circle).


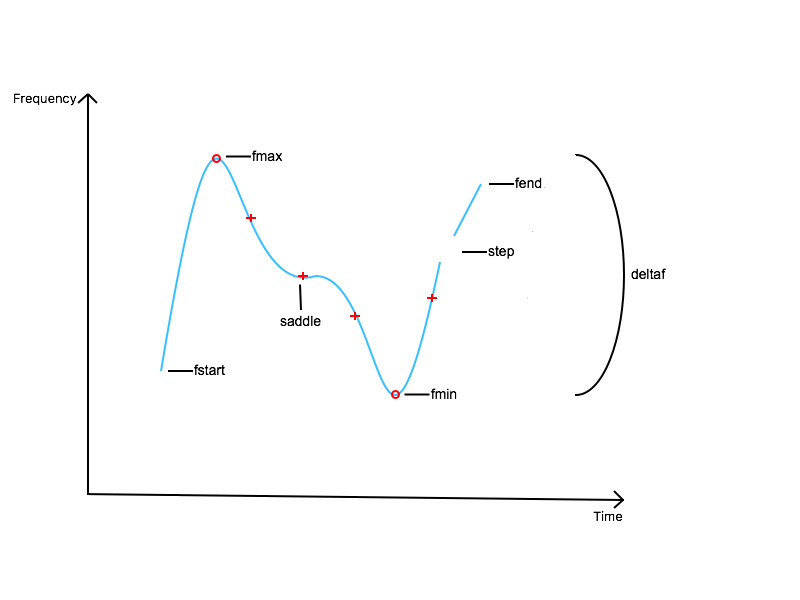


Figure S2: Whistle Characteristics (fstart = start frequency, fend = end frequency, fmax = maximum frequency, fmin = minimum frequency, deltaf = delta frequency (fmax-fmin), inflection points are + and local extrema are O).


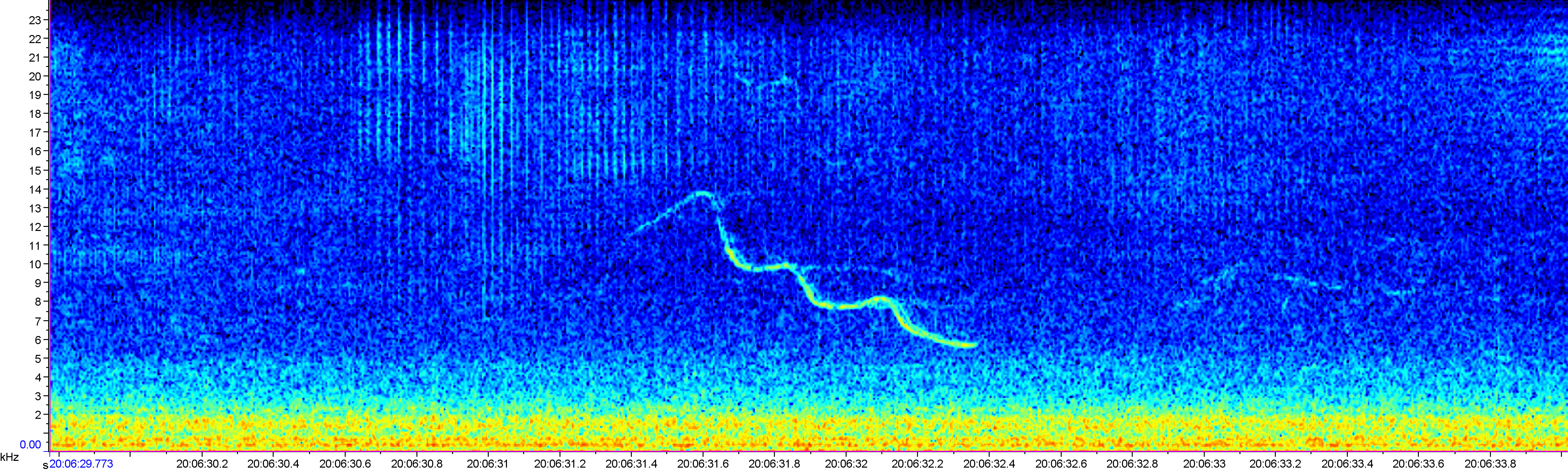


Figure S3: Example spectrogram of a dolphin whistle.

a)


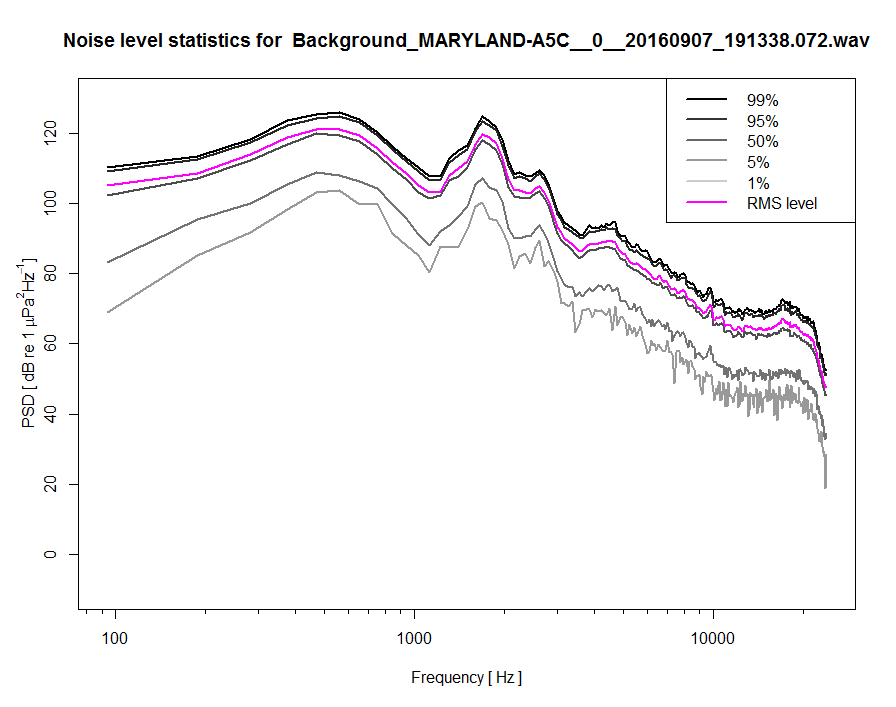


b)


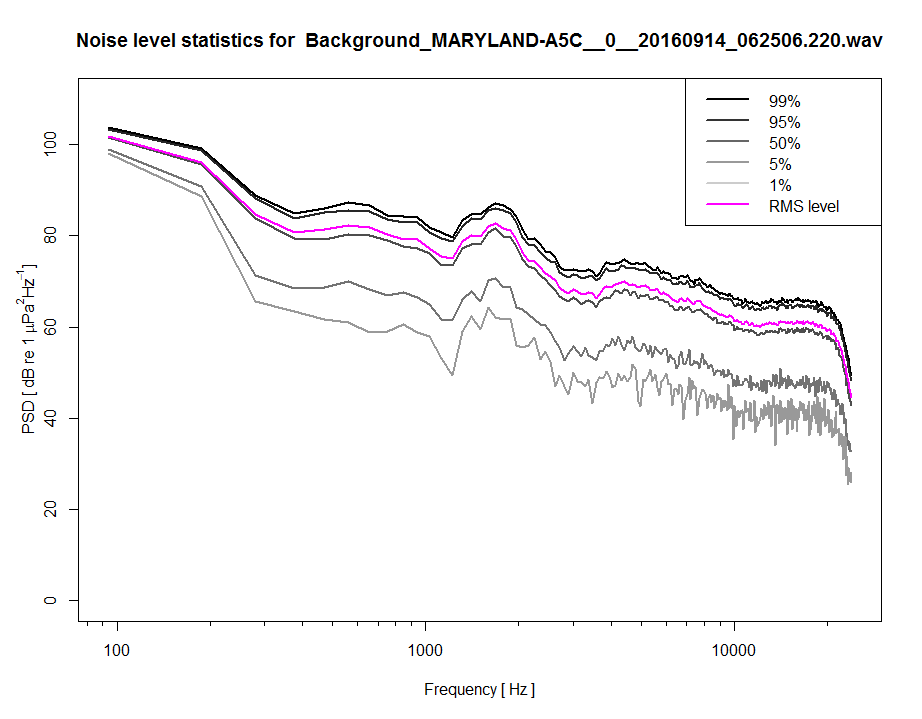


Figure S4: Power spectral density of the loudest (7^th^ September 2016) and quietest (14^th^ September 2016) ambient noise levels contemporaneous with selected high quality whistles with the percentiles and root-mean-square (RMS) sound pressure levels shown.


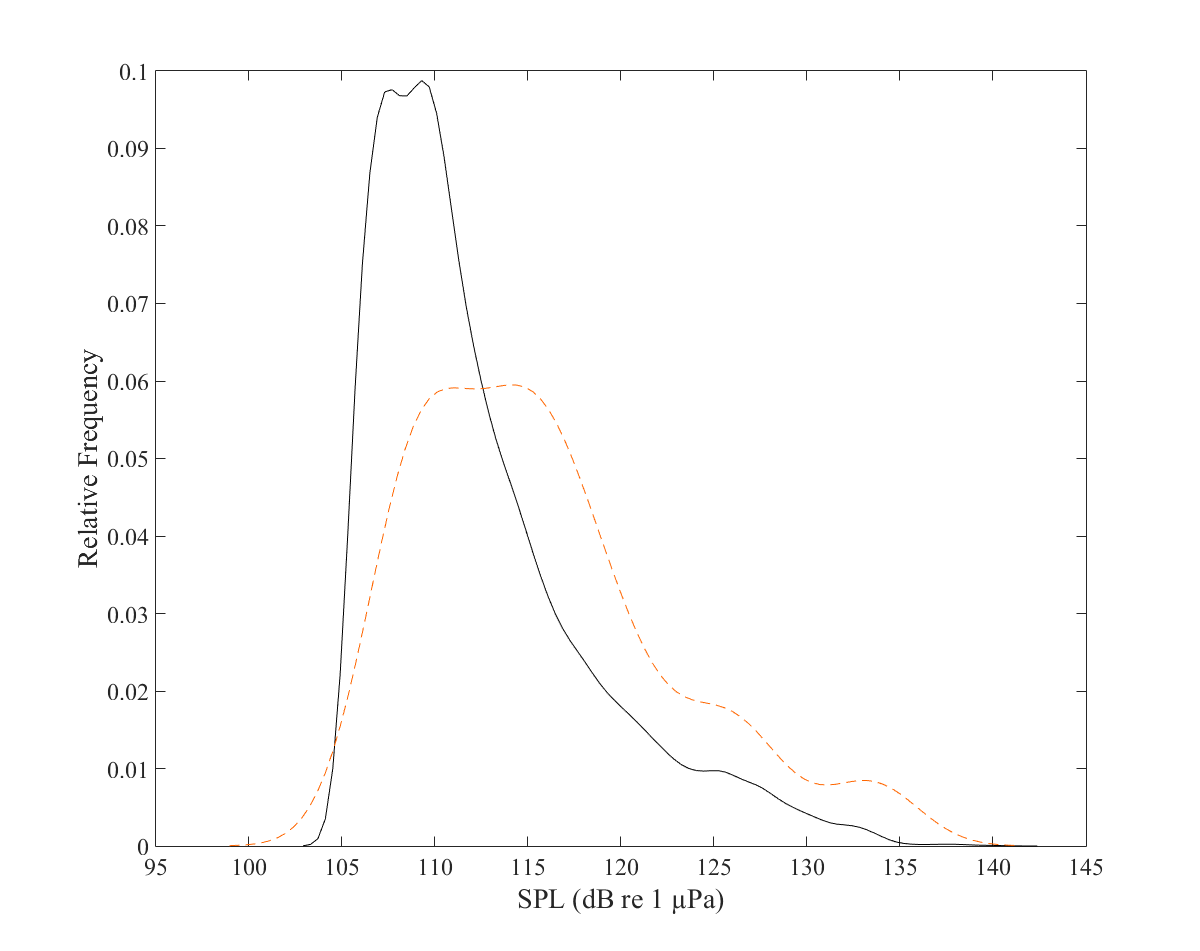


Figure S5: Ambient noise levels during the entire deployment period July-September 2016 (black line) and contemporaneous with selected high-quality whistles (dotted red line).
